# Misinterpretation of an inflammatory FDG uptake in a patient treated for Hodgkin lymphoma: a case report

**DOI:** 10.22038/AOJNMB.2022.66011.1457

**Published:** 2023

**Authors:** Alberto Nieri, Luca Urso, Matteo Caracciolo, Maria Ciccone, Licia Uccelli, Corrado Cittanti, Antonio Cuneo, Mirco Bartolomei

**Affiliations:** 1 Nuclear Medicine Unit, University Hospital of Ferrara, Ferrara, Italy; 2 Department of Translational Medicine, University of Ferrara, Ferrara, Italy; 3 Hematology Unit, University Hospital of Ferrara, Ferrara, Italy

**Keywords:** Hodgkin Lymphoma, ^18^F-FDG PET/CT, Therapy response, Pitfall, Biopsy

## Abstract

Hodgkin Lymphoma (HL) is a malignancy involving lymph nodes and lymphatic system. [^18^F]F-FDG PET/CT (FDG-PET) imaging is routinely used for staging, to assess early chemotherapy response (interim FDG-PET), at the end of treatment (EoT FDG-PET) and for the identification of disease recurrence.

We present a case of a 39-year-old man treated for HL. FDG-PET scans performed after first line therapy (both Interim PET and at the end of therapy) demonstrated a persistent and significant mediastinal FDG uptake. The patient was treated with a second line therapy but the FDG-PET uptake did not change. After board discussion a new surgical, thoracoscopy-guided biopsy was performed. Histopathology demonstrated a dense fibrous tissue with occasional chronic inflammatory infiltrates.

Persistent FDG-PET positivity may suggest refractory or relapsed disease. However, occasionally, non-malignant conditions are responsible for a persistent FDG uptake, not related to primary disease. An accurate evaluation of clinical history and previous imaging exams is mandatory for clinicians and others experts to avoid misinterpretations of FDG-PET results. Nevertheless, in some cases, only a more invasive procedure, such as a biopsy, may finally lead to a definitive diagnosis.

## Introduction

 Hodgkin lymphoma (HL) is a hematologic malignancy involving mainly lymph nodes and the lymphatic system. Currently, HL has a favourable prognosis, with respectively 90% and 80% patients alive and disease free after a 6-year follow-up (1-4).

 Conventional imaging – CT in particular - can provide anatomical details and define the extension of the disease in patients with HL. However, only ^18^F-FDG PET/CT (FDG-PET) can assess the metabolic activity and, therefore, reveal the malignant and eventually aggressive nature of a lesion. Several studies indicate that FDG-PET is more accurate than CT and it is recommended at diagnosis and for therapy response assessment in patients with HL and other FDG-avid lymphomas (5, 6).

 FDG-PET is routinely used for staging (baseline FDG-PET), to assess the response after 2 cycles (interim FDG-PET), at the end of treatment (EoT FDG-PET) and for the identification of possible relapses (7–9). In this context, the 5 points Deauville scale (DS) is a recognized and validated tool to guide the clinician into the decision of the best therapeutic plan. In particular, the persistence of an intense uptake at interim FDG-PET, if located in a site of disease at the baseline study or in new sites, may suggest a treatment intensification (9, 10). 

 We report here an uncommon case of a misleading persistence of FDG uptake in a patient with HL.

***Case Report***

 In July 2020, a 39-year-old man was admitted at the outpatient clinic of the Haematology Unit for persistent itching and night sweats. Therefore, a contrast enhanced CT scan (CeCT) was performed and revealed mediastinum lymph-nodes enlargement, with the largest one in the anterior mediastinum (7×14×6 cm^3^). Baseline FDG-PET showed intense uptake in correspondence of the large lymph node mass located in the anterior mediastinum (SUV_max_=18) and of other lymph nodes located near the aortic arch.

 The subsequent biopsy of the anterior mediastinum mass demonstrated fibroadipose tissue including a lymphoid infiltrate consisting of atypical elements of large size, referable to Hodgkin and Reed-Sternberg cells (CD30 + /CD15 + /PAX5 +/IRF4 +). The final diagnosis was classic Hodgkin Lymphoma (cHL), nodular sclerosis variant, stage IIA (B-symptoms were mild and not persistent), EORTC and GHSG intermediate risk for the presence of bulky disease and elevated erythrocyte sedimentation rate.

 According with current guidelines, from august to October 2020 the patient was treated with two cycles of doxorubicin, bleomycin, vinblastine and dacarbazine (ABVD) chemotherapy.

 After two ABVD cycles, interim FDG-PET evaluation demonstrated a significant dimensional reduction of the previous findings, with the disappearance of the uptake at lymph nodes located near the aortic arch but the persistence of a core of focal uptake limited at the anterior mediastinum (SUV_max_=11). No evidence of new disease localizations was found. Despite the DS of 4 at the interim PET, two additional ABVD cycles were preferred over early intensification of treatment. No severe toxicity has been reported other than grade 3 neutropenia that resolved with granulocyte growth factor (G-CSF).

 EoT FDG-PET evaluation, performed in December 2020, showed the persistence of the previously reported uptake (SUV_max_=13; DS=4) and CeCT demonstrated the persistence of the anterior mediastinum lesion.

 Consequently, an intensification of treatment was proposed and the patient was treated with three cycles of Bendamustine, Gemcitabine and Vinorelbine (BeGEV) from January 2020 to March 2021. As expected, the patient developed haematology toxicity that was manageable and did not require treatment interruction. After three cycles of BeGEV, a FDG-PET evaluation was performed (March 2021) with no significant changes in comparison with the previous scan (SUV_max_=14.6). Therefore, the finding was considered by the nuclear medicine physician more likely referable to a HL residual localization.

 However, due to the persistent uptake without significant changes between interim PET and the exam performed after the high dose chemotherapy and considering that the patient was completely asymptomatic, a new mediastinum biopsy of the residual mass in correspondence of the maximum FDG uptake was proposed to the patient in order to confirm refractory disease. A surgical, thoracoscopy-guided biopsy revealed a dense fibrous tissue with occasional chronic inflammatory infiltrates, with macrophage component CD68+. No evidence of HL was found.

**Figure 1:**
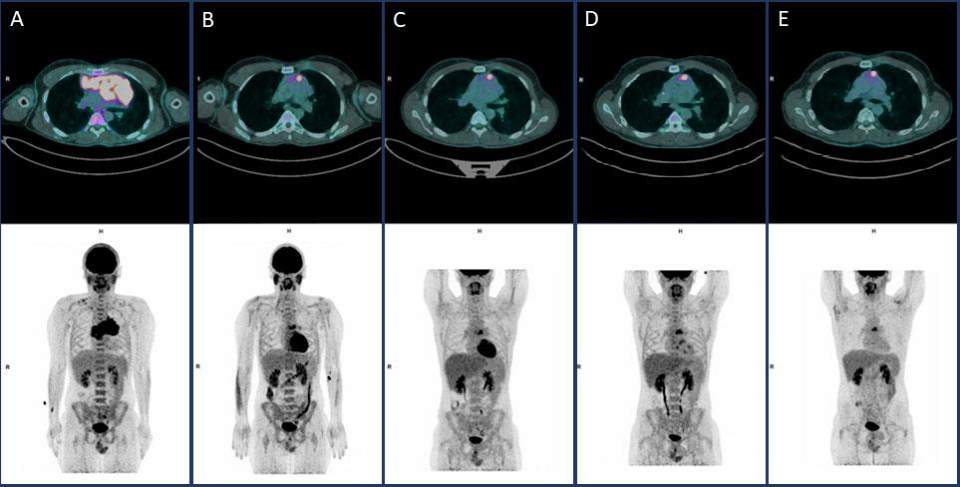
FDG-PET findings at [**A**] Baseline FDG-PET, [**B**] Interim FDG-PET (ABVD), [**C**] EoT FDG-PET (ABVD), [**D**], after three cycles BeGEV FDG-PET and [**E**] Follow Up after three months from last chemotherapy

 Therefore, patient was judged in complete remission and no further treatment has been scheduled, but a close clinical follow-up was established. A new FDG-PET scan performed 3 months later (July 2021) showed no changes in comparison to the previous exams (Figure 1).

 The patient’s clinical conditions were good, without any sign of disease relapse. Thus, a clinical and FDG-PET follow-up every six months was scheduled. No changes in patient’s health status were observed. The 2 most recent FDG-PET scans were performed 10 (January 2022) and 15 months after the biopsy (July 2022). Both the exams demonstrated the persistence of the known uptake roughly unchanged (SUV_max_ 9.4 and 10.8 respectively). In consideration of patient’s clinical conditions and imaging results a new biopsy was not indicated and follow-up every six months will be continued. 

## Discussion

 We report the case of patient with HL and intense and persistent FDG uptake after ABVD and high dose therapy. FDG-PET is recommended to assess treatment response in patients with HL (10). Many studies showed that a rapid uptake reduction occurring after two or three cycles into a planned course of chemotherapy is predictive of an excellent prognosis. Conversely, patients with persistent uptake on interim FDG-PET scans have a poorer prognosis, with progression-free survival (PFS) and overall survival (OS) rates dependent on histology and disease stage (11). Many scores have been proposed to standardize treatment response evaluation and, currently, DS is a recognized useful guide for the clinician in the therapy management. Although initially developed for interim reporting, the DS is currently also recommended to evaluate the response assessment at the end of treatment by the 2018 guidelines (10).

 However, due to the evidence that FDG uptake can occasionally be non-specific, the management of a persistent residual FDG uptake can be challenging and should be carefully evaluated in order to avoid overtreatments. Several literature data report no correlation between persistent FDG uptake and primary disease in patients treated with chemotherapy or immunotherapy for HL, with an overall 23.1% of false positive (FP) in EoT FDG-PET. In these cases histopathology revealed either benign (inflammatory) and malignant conditions, such as granulomatous disease (12), sarcoidosis (13), thymus hyperplasia (14) and many others (15).

 To identify FP findings some authors evaluated the role of SUV_max_ parameter. Zinzani et al in 2007 described a significantly higher SUV_max _in true positives (median values 5.95, range 3.5-26.9) than in FP (median SUV_max_=2.90, range, 1.4-3.3) FDG-avid mediastinum residual masses that underwent histological confirmation (16). 

 Conversely, SUV_max_ was persistently high in our case, emphasizing that SUV_max_ is not a reliable parameter for discriminating the nature of a residual uptake.

 Some studies suggested new methods to try to better characterize these uncertain findings. Both in vitro and in vivo evidences demonstrated that various malignant neoplasms exhibit an increase in FDG uptake or SUV over time compared to inflammatory tissue. Therefore, some authors suggest the adoption of a dual scan protocol (after 60 and 120 minutes) (17). This scheme may allow to distinguish between lymphomatous processes and other causes (especially inflammatory) evaluating SUV_max_ variations between the 2 scans. This type of approach is barely usable in routine clinical practice; however it may solve the clinical dilemma in some cases.

 Another possibility could be to identify alternative tracers that could better characterize residual masses. In this scenario ^18^F-Fluoro-deoxy-thymidine PET ([^18^F]F-FLT-PET) seems to be a promising method for early therapeutic monitoring patients treated for FDG-avid lymphomas (18). A pilot study by Mena et al reported that [^18^F]-FLT-PET could be useful to differentiate between residual malignant disease and benign processes, but further studies are needed (19).

 However, the present case suggests that, if the clinical index of suspicion is low, a more invasive procedure should be considered in order to discriminate the lesion’s nature and to address the subsequent therapy program. In case of a FP, a correct follow-up should be planned, reserving new treatment in case of a relapse confirmed by surgical biopsy. This approach could be preferable, especially in case of young people and children, to avoid potential overtreatments in a potentially curable disease (20).

## Conclusion

 An accurate evaluation of clinical history and previous imaging exams is mandatory to avoid misinterpretations, particularly in case of low clinical suspicion. We suggest investigating uncertain FDG-PET findings in HL patients recurring to a different scan protocol, or using another radiotracer. In case of inconclusive results, a biopsy should be considered, in order to avoid potential overtreatments and schedule the correct follow-up.

## Conflicts of Interest

 The authors declare no conflict of interest.
